# Research Progress of Dual-energy CT in Diagnosis and Evaluation of Curative Effect of Liver Cancer: A Review

**DOI:** 10.2174/0115734056335472250310085121

**Published:** 2025-04-17

**Authors:** Mingtai Cao, Yumiao Qiao, Xukun Gao, Xinyi Liu, Airu Yang, Rui Fan, Boqi Zhou, Bin Huang, Yuntai Cao

**Affiliations:** 1 Department of Radiology, Affiliated Hospital of Qinghai University, Xining, China; 2 Department of Medical Ultrasound, Tongji Hospital, Tongji Medical College, Huazhong University of Science and Technology, Wuhan, China

**Keywords:** Hepatocellular carcinoma, Dual-energy computed tomography, Accurate diagnosis, Efficacy assessment, Lymph node metastasis, Image Reconstruction, Microvascular Invasion

## Abstract

Primary liver cancer is the sixth most common cancer and the third leading cause of cancer deaths worldwide, with over 900,000 new cases and more than 800,000 deaths annually. Conventional imaging techniques have improved the diagnosis and assessment of treatment response in patients with Hepatocellular Carcinoma (HCC), but they have many limitations. Introducing Dual-Energy Computed Tomography (DECT) into clinical practice offers an opportunity to address these issues. DECT has unique advantages in diagnosing and evaluating the efficacy of HCC treatment. It can provide quantitative information on various substances and, through multi-parameter and quantitative parameter analysis, can be used for early detection of HCC, identification of benign and malignant lesions, and monitoring of lymph node metastasis and Microvascular Invasion (MVI). Additionally, DECT provides valuable information for evaluating therapeutic efficacy. This review covers the imaging principles of DECT, including its basic principles, scanner design modes, and Image Reconstruction (IR) techniques. It then describes the research progress of DECT in diagnosing HCC and evaluating treatment efficacy. Finally, it briefly discusses some limitations of DECT and its future development directions.

## 1 INTRODUCTION

Primary liver cancer is the sixth most common cancer and the third leading cause of cancer-related death worldwide, with over 900,000 new cases diagnosed annually. It is projected that by 2025, more than 1 million new cases of Hepatocellular Carcinoma (HCC) will be identified each year [1]. Despite advancements in prevention, diagnosis, and treatment, the five-year survival rate for HCC remains low at only 20%, primarily due to its high recurrence and metastasis rates [2, 3]. Accurate diagnosis of suspected liver lesions is crucial for developing effective treatment plans, which can significantly enhance patient survival rates. Imaging is the cornerstone of diagnosing suspicious liver masses and is essential for the diagnosis, staging, treatment planning, and efficacy assessment in patients with suspected or confirmed HCC [4]. With ongoing advancements in medical imaging technology, new modalities such as Dual-Energy Computed Tomography (DECT) have been introduced, offering enhanced accuracy in the evaluation and assessment of HCC.

## 2 BASIC PRINCIPLES AND IMAGING TECHNIQUES OF DECT

DECT was first proposed in 1973 and further explored by Alvarez and Marcovski in 1976 [5, 6]. However, early technical limitations, such as the necessity for two separate data acquisitions, issues with temporal and spatial alignment, low tube voltages, and high radiation exposure, prevented DECT from being used in clinical practice at that time [7]. With technological advancements, DECT was finally introduced into clinical settings in 2006 [7, 8]. DECT enhances image quality by acquiring scans at two different X-ray tube energies, typically 80 kVp and 140 kVp, either simultaneously or nearly simultaneously. The differing attenuation of these two X-rays is measured using a standard detector to define, differentiate, and quantify the composition of various tissues or lesions [9].

### 2.1 Different Ttechniques for DECT

Dual-Source Dual-Energy CT (DS-DECT): This technique uses two independent X-ray tubes and corresponding detectors, each operating at different tube voltages. The X-ray tubes are mounted orthogonally to the frame at an angle of approximately 90° relative to their detectors [10]. High-energy tubes operate at 120 kVp or 140 kVp, while low-energy tubes typically operate at 80 kVp or 100 kVp. For larger obese patients, an 80 kVp setting is not recommended, as increasing the voltage setting to 100 kVp improves image quality in such cases [7].

Rapid Kilovoltage-Peak Switching Dual-Energy CT (KVS-DECT): This method uses an X-ray tube and detector that rapidly alternates between high and low energy voltages (80 kVp and 140 kVp, with an interval of about 0.5 ms) [10, 11]. It performs two scans at each position to produce two sets of data [11]. Despite slightly slower rack rotation times, KVS-DECT provides excellent temporal and spatial alignment and is more cost-effective than DS-DECT [11]. The ability to obtain data at two different energies from a single scan provides rich energy resolution information.

**Fig. (1):**
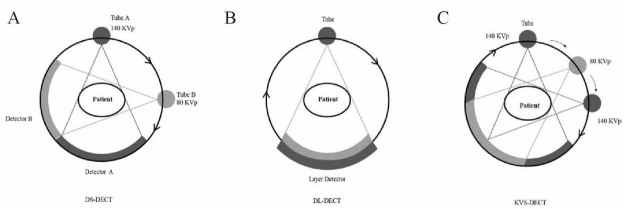
Different techniques for DECT. (**A**) DS-DECT uses two independent X-ray tubes and their corresponding detectors, operating at different tube voltages. (**B**) DL-DECT uses two detector layers to complete the scan. This “sandwich layer” detector consists of two layers of scintillators. (**C**) KVS-DECT consists of an X-ray tube and a detector that rapidly alternates between high and low energy voltages.

Dual-Layer Detector Dual-Energy CT (DL-DECT): This technique uses two detector layers to complete the scan. The “sandwich layer” detector consists of two layers of scintillators: the upper scintillator, containing ZnSe crystals, primarily absorbs low-energy photons, while the lower scintillator, made of Gd_2_O_2_S material, absorbs high-energy photons that pass through [12]. This configuration not only improves the energetic resolution of the imaging but also allows for more precise tissue composition analysis, enhancing contrast and providing richer diagnostic information (Fig. **1**).

The DECT system can generate various types of Image Reconstructions (IR) through post-processing, including Virtual Monochromatic Images (VMI), Virtual Non-Contrast-enhanced (VNC) images, material density reconstructions (such as iodine maps), and energy spectrum curves. These advanced imaging techniques play a crucial role in diagnosing and treating HCC.

### 2.2 Virtual Monochromatic Images

VMI technology simulates the X-ray attenuation value of an object at specific single energy levels by analyzing datasets obtained from high and low-energy scans [13]. A post-processing workstation generates VMI 40 to 140 keV, allowing for detailed observation and analysis of the morphological characteristics of lesions [14]. Lower energy levels provide higher tissue contrast, while higher energy levels reduce tissue contrast enhancement but are less affected by beam hardening and photon starvation effects [15]. Monoenergetic images at 70 keV demonstrate better image quality compared to conventional multicolor 120-kVp images at the same radiation dose [16]. The optimal energy range for monochromatic images is typically between 40 and 55 keV [17-19], though image quality can be compromised by increased low-energy noise and patient size [20]. Advanced noise-optimized VMI reconstruction algorithms can mitigate these limitations to some extent.

### 2.3 Virtual Non–contrast Images

VNC images are created from contrast-enhanced scans by virtually subtracting iodine, thus simulating a non-contrast image [21]. Studies have shown that replacing True Non-Contrast (TNC) images with VNC images can reduce radiation dose [22]. VNC images are recommended as an alternative to TNC images for characterizing HCC, with reported accuracy rates between 87.5% and 92.9% [23, 24]. However, their clinical use is currently limited due to susceptibilitythe sensitivity to artifacts and reduced sensitivity to small calcified foci [25]. Future advancements in energy spectrum separation and material decomposition techniques, such as multi-material decomposition algorithms, are expected to improve the appearance and attenuation stability of VNC images.

### 2.4 Separation and Quantitative Analysis of Substances

DECT enables the quantification of different preselected materials (such as iodine, fat, and calcium) to generate corresponding images, like iodine maps [15]. Iodine mapping is particularly useful for detecting and characterizing iodine-containing lesions and identifying areas of ischemia or hemorrhage [26]. In patients with chronic liver disease, accurately identifying and characterizing liver masses is crucial for effective patient management [27]. Kaltenbach *et al*. [28] demonstrated that DECT Arterial Phase (AP) iodine quantification significantly improves the diagnostic performance for differentiating hepatic neuroendocrine tumor metastasis from HCC compared to conventional attenuation measurements. Another study showed that DECT-generated material decomposition parameters are effective in distinguishing malignant or potentially malignant nodules from benign nodules in cirrhotic patients [29]. Recently, DECT-based iodine quantification has emerged as a promising quantitative imaging biomarker for improving lesion detection and monitoring tumor response to treatment [30-33]. However, the accuracy of iodine quantification can be affected by different scanners and patient body types [34].

### 2.5 Energy Spectrum Curves

**Fig. (2):**
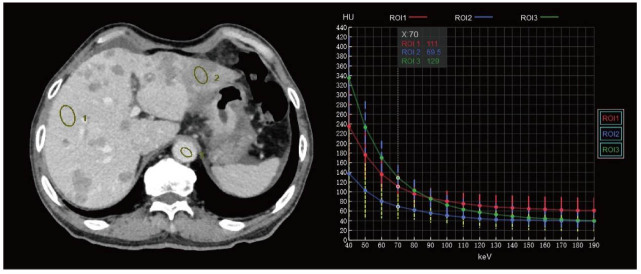
Energy spectrum curves. By placing multiple ROIs on the image and plotting their energy spectrum curves, ROIs can be compared to determine whether they are composed of similar or different materials, thereby identifying different lesions.

Energy spectrum curves reflect the attenuation properties of different substances or structures. By analyzing these spectral curves, it is possible to obtain the average Computed Tomography (CT) value and standard deviation for each energy point from 40 to 140 keV [35]. Each substance has a unique energy spectrum curve. At a specific monochromatic energy level, the average attenuation value of a Region of nterest (ROI) depends on the materials present in the ROI and their quantities. By placing multiple ROIs on an image and plotting their spectral decay curves, these curves can be compared to determine if the ROIs are composed of similar or different materials, thus allowing assessment of their potential chemical composition (Fig. **2**) [36]. Virtual spectral curves generated by DECT are an effective method for differentiating focal liver lesions. The slopes and characteristics of the virtual spectral curves for HCC, hepatic hemangiomas, metastases, and cysts differ significantly. Therefore, quantitative analysis of DECT energy spectral curves can enhance the sensitivity of liver tumor identification [37].

## 3 RESEARCH PROGRESS OF DECT IN LIVER CANCER DIAGNOSIS

Since DECT's introduction into clinical practice, extensive research has been conducted on its application in the early diagnosis of liver cancer, Microvascular Invasion (MVI), lymph node metastasis, and other aspects. Numerous studies have confirmed the critical value of DECT as an auxiliary tool for accurately diagnosing HCC. This section provides a brief overview of the research progress of DECT in diagnosing HCC.

### 3.1 Definite Diagnosis of Liver Cancer

**Fig. (3):**
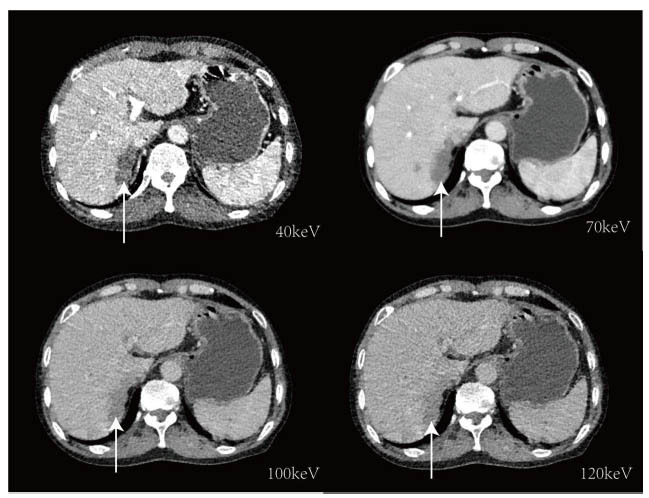
Comparison of different VMIs. Higher contrast can be obtained by low energy VMI (40 keV) in DECT after intravenous contrast enhancement, but with greater noise. Lower contrast can be obtained from high energy VMI (120 keV). The best CNR occurs at 70keV.

DECT's ability to differentiate and quantify substances enhances liver cancer diagnostic performance, as demonstrated in numerous clinical applications [38]. Low-energy VMIs are characterized by significant iodine attenuation, resulting in higher contrast and improving the early diagnosis of HCC [39]. Sudarski *et al*. [40] found that the peak Contrast-to-Noise Ratio (CNR) for liver parenchyma and liver lesions occurs at 70 keV due to higher attenuation and lower image noise. However, high image noise adversely affects VMI datasets below 70 keV, limiting clinical application (Fig. **3**) [41].

The novel IR technique, Deep Learning Image Reconstruction (DLIR), integrates Deep Convolutional Neural Networks (DCNN) into IR algorithms. The DCNN is trained using high-quality image datasets to effectively differentiate between noise and signal. Both qualitative and quantitative assessments have demonstrated that DLIR enhances vessel clarity, CNR, and lesion clarity in VMI and ID images obtained from abdominal contrast-enhanced DECT when compared to traditional hybrid iterative reconstruction methods [42]. Deep Learning (DL)-based spectral CT imaging represents an innovative approach to KVS-DECT, incorporating cascaded DL reconstruction techniques. This method addresses the issue of incomplete views in the sinusoidal map space and enhances image quality within the image space by employing a DCNN trained on fully sampled dual-energy data acquired through double kilovolt rotation. The present study demonstrates that Iodine Density (ID) maps derived from DL-based spectral CT imaging exhibit superior clinical utility for evaluating arterial vascular distribution in HCC [39]. Li *et al*. [43] extracted DL features and handcrafted features from VMIs and biphasic material composition images to build a DL model to predict macrotrabecular-massive HCC using the UCTransNet network. The results indicate that the model demonstrates high predictive accuracy for Macrotrabecular-Massive HCC, with the Area Under the receiver operating characteristic Curve (AUC) values of 0.91, 0.87, and 0.89 for the training set, internal validation set, and external validation set, respectively.

DLIR shows promise in reducing image noise and improving the detectability of low-energy VMIs [44]. DL-based 40 keV images have shown better lesion diagnostic performance with noise reduction [45]. The noise-optimized VMI algorithm combines the greater iodine attenuation of lower-energy monoenergetic images with the low noise of higher-energy monoenergetic images. This results in higher CNR obtained at lower energy levels, with the noise-optimized VMI algorithm (50 keV) dataset providing superior image quality and diagnostic accuracy for detecting blood-rich liver lesions less than 1 cm in diameter compared to VMI or linearly-blended reconstruction [17]. These studies indicate that IR algorithms can significantly improve the diagnostic performance of DECT.

However, some studies have indicated that DECT appears to be less effective than enhanced Magnetic Resonance Imaging (MRI) yet more effective than plain spiral CT in identifying HCC lesions. The sensitivity of MRI with extracellular contrast agents and MRI with gadoxetic acid for detecting HCC foci in patients with cirrhosis was reported to be 78.5% and 69.7%, respectively [46]. Both DECT and MRI exhibit a sensitivity of 69% and a diagnostic specificity of 60% for the detection of small HCC lesions [17]. Combined with geodetic acid-enhanced MRI, the specificity of diffusion-weighted imaging for detecting small HCC (less than 1 cm) was 90.0% [47]. De Cecco *et al*. [17] suggested that the application of a noise optimization algorithm has the potential to improve the diagnostic specificity of DECT for detecting blood-rich liver lesions by 11%. Yoon JH *et al*. [48] found that DECT low single-energy images (50keV) significantly improved the visualization rate of lesions, and in combination with their pathological results, found that the diagnostic specificity (93.10% *vs*. 72.41%) and accuracy (90.82% *vs*. 76.53%) of DECT were higher than those of plain spiral CT. Further optimization of DECT quantitative parameters and imaging modalities in the future is expected to improve the HCC detection rate.

#### 3.1.1 Extent of Infiltration

Portal Vein Thrombosis (PVT) may indicate a hypercoagulable state and often forms in malignancies [15]. Approximately 44% of HCC patients have malignant PVT caused by tumor invasion of the portal vein or its tributaries. Identifying benign and malignant thrombi is crucial for prognosis, staging, and treatment planning. Malignant thrombosis may exclude treatment methods such as surgical resection and orthotopic liver transplantation [49]. While histologic diagnosis is standard, noninvasive techniques such as MRI, doppler ultrasound, and CT imaging also play vital roles [15].

In conventional enhanced CT, the presence of thrombus enhancement in the late AP is specific for malignant thrombus. However, the technique is limited by difficulties in placing the ROI [15, 49]. DECT's venous phase parameters aid in thrombus detection, and iodine maps obtained from the late AP dataset allow qualitative assessment of thrombus and measurement of iodine uptake in the PVT. Accurately distinguishing between benign and malignant PVT with a critical value of 0.9 mg/mL has high diagnostic efficacy and accuracy [49]. Additionally, datasets obtained from DECT's venous phase acquisition distinguished benign from malignant PVT with an ID of 1.14 mg/mL [50].

These studies underscore DECT's significance in diagnosing malignant PVT, suggesting it can be an effective complementary tool for noninvasively assessing HCC infiltration preoperatively. DECT is expected to become one of the primary tools for accurately identifying malignant PVT in the future.

#### 3.1.2 Lymph Node Metastasis

Lymph node metastasis is a significant prognostic factor in HCC, with an incidence ranging from 4.2% to 8.4%, and is often missed or misdiagnosed [51, 52]. Surgical clearance of regionally invaded lymph nodes can improve patient survival, making accurate assessment and identification of metastatic lymph nodes essential [52, 53]. Current methods for lymph node evaluation, such as ultrasound, CT, and MRI, have low sensitivity and specificity [54]. DECT offers a new method for assessing lymph node metastasis in malignant tumors.

Mo *et al*. [55] found that the maximum short-axis diameter of lymph nodes was less accurate in differentiating metastatic from non-metastatic lymph nodes in HCC. DECT parameters and Normalized Iodine Concentration (NIC) in the AP were more accurate for diagnosing HCC metastatic lymph nodes, with a sensitivity and specificity of over 80%. The best diagnostic performance was achieved with an AP NIC value of 0.175. Metastatic lymph nodes had lower Iodine Concentration (IC) and NIC than non-metastatic lymph nodes, indicating reduced blood supply. However, Zeng *et al*. [56] found higher iodine levels in metastatic lymph nodes compared to non-metastatic ones, suggesting the need for further studies to explore these differences. Combining the maximum short-axis diameter with IC, NIC, or the slope of the energy spectrum (λHU) values provided superior diagnostic performance compared to using any single parameter alone.

These studies highlight that DECT is useful for the noninvasive quantitative assessment of metastatic lymph nodes, which is crucial for increasing patient survival and improving prognosis. However, these studies excluded lymph nodes with a maximum short-axis diameter of less than 4-5 mm due to the uncertainty of ROI measurements. Additionally, DECT images were not compared with MRI, and the studies included small patient samples without multicenter validation. Thus, multicenter, large-sample studies are needed to verify these findings.

#### 3.1.3 Microvascular Invasion

MVI is an independent risk factor for intrahepatic metastasis and postoperative recurrence of HCC [57]. Early and accurate prediction of MVI before surgery can help doctors develop more appropriate treatment plans, significantly improving patient prognosis.

DL-DECT is effective for the preoperative prediction of MVI in alpha-fetoprotein negative HCC. The ID Normalized Iodine Density (NID), and Effective atomic number (Eff-Z) in the AP, as well as the ID in the delayed phase, were significantly higher in the MVI-positive group than in the non-MVI group [58]. Yang *et al*. [57] found that NIC was significantly higher in the MVI group than in the non-MVI group. Other studies have shown that peritumoral enhancement is associated with an increased risk of MVI [59]. Chou *et al*. [60] demonstrated that NIC in the peritumoral layer was an independent predictor of MVI. This suggests that MVI status can be predicted by analyzing NICs in both the intratumoral and peritumoral regions, as well as in the peritumoral region alone.

These findings suggest that DECT iodine quantification parameters can reflect the neovascularization and microcirculatory perfusion of HCC lesions during MVI. DECT offers a new method for assessing MVI, enabling accurate prediction and determination of MVI status in HCC patients, thereby aiding in the development of more precise treatment plans.

## 4 DIFFERENTIAL DIAGNOSIS

**Fig. (4):**
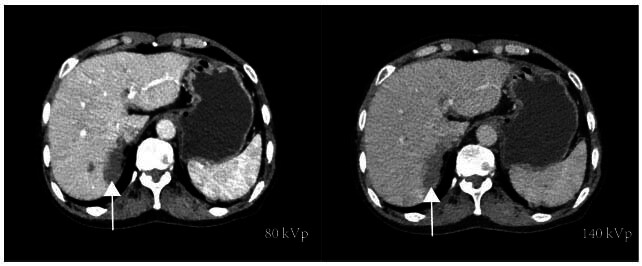
Original dual-energy images. Low kVp imaging can enhance lesion contrast and make it easier to detect HCC lesions. However, the main disadvantage is a significant increase in noise levels.

DECT offers notable advantages in liver imaging, such as improved lesion saliency and diagnostic accuracy. Low-kVp imaging enhances the attenuation differences between lesions and liver parenchyma, making it easier to detect lesions in the advanced hepatic arterial stage. However, a major disadvantage is a significant increase in noise level [7, 61] (Fig. **4**). VMI at 50-55 keV can improve liver lesion definition, CNR, and the detection of blood-rich tumors [62]. Additionally, monoenergetic datasets combined with iodine density images enhance liver lesion detection due to iodine's low K-edge value (33.2 keV), which facilitates its depiction at lower energy states [63].

DECT imaging combined with alpha-fetoprotein has shown high sensitivity (98.1%) and accuracy (89.1%) in identifying HCC and focal nodular hyperplasia [64]. In another study, the NIC within the lesion and the lesion-to-normal parenchyma iodine ratio (LNR) effectively distinguished focal nodular hyperplasia from HCC, with a sensitivity and specificity of 100% when the LNR threshold value was 4.33 [65]. LNR, NIC, and iodine uptake were higher in HCC compared to neuroendocrine tumors [28]. Additionally, necrotic HCC can be differentiated from abscesses by higher NIC and LNR values [55]. Quantitative DECT analysis can also distinguish liver abscesses from liver metastases, as metastases have significantly higher CT values, Eff-Z, and lower fat concentrations than liver abscesses on VMI (40-140 keV) [66]. KVS-DECT iodine quantitative image analysis can accurately distinguish hepatic hemangioma from HCC since small hemangiomas have higher iodine concentrations than small HCC [28].

DECT imaging of hepatic lesions in the portal venous phase reveals four lesion types with different baseline levels of energy spectral curves. Analyzing the curve slope can help differentiate between hemangiomas, HCC, metastases, and simple cysts, making it an effective diagnostic indicator, especially for distinguishing hemangiomas and cysts, with a diagnostic specificity of 100% [67]. Recently, the introduction of noise-optimized reconstruction algorithms has addressed the problem of increased noise, providing higher CNR and diagnostic efficiency for better assessment of liver lesions through lower VMI keV [68, 69]. Large (11 mm) and high-contrast (45 HU) lesions are easily detected on conventional multi-energy images. However, detection rates donot significantly improve using VMI (40 keV), which has the highest iodine contrast and better improves the detection rate of small metastatic lesions (<1 cm) [70]. Therefore, combining VMI (40 keV) with conventional multi-energy images or VMI (70 keV) at higher energies is recommended for accurate lesion detection [17, 71].

The DL reconstruction algorithm improves image quality by suppressing image noise and preserving structural details. Iodine maps generated by this algorithm outperform VMI (70 keV) in displaying iodine contrast, significantly improving the detection rate of CNR and HCC lesions [72]. However, for smaller volumes or lower iodine concentrations, the effect may not be satisfactory [39].

These studies demonstrate that DECT has significant advantages in the differential diagnosis of HCC. Effective differentiation between HCC and benign liver lesions can be achieved through quantitative iodine analysis, VMI, and energy spectrum curves.

## 5 EVALUATION OF CURATIVE EFFECT

Despite various treatment strategies, the five-year survival rate for HCC remains low in developing countries [2, 3]. Most patients are unsuitable for surgical treatment upon diagnosis, necessitating early monitoring for patients with advanced disease [73, 74]. Various therapies can help slow disease progression [75]. Traditional assessment methods, such as the response evaluation criteria in solid tumors, version 1.1, primarily focus on changes in tumor size and may not accurately reflect treatment efficacy [15]. DECT offers an advantage by assessing changes in the vascular supply to the tumor through monitoring iodine uptake, allowing for a more rapid and accurate evaluation of treatment response [76].

### 5.1 Anti-angiogenic Drugs

Tumor angiogenesis is a crucial factor in tumor growth and metastasis. A common therapeutic approach involves using agents that target tumor vasculature. DECT can assess the effectiveness of these therapies by measuring iodine levels in tumor tissue [77]. Changes in iodine uptake in patients with advanced HCC treated with drugs such as sorafenib can be a useful indication of treatment response [78]. Anti-angiogenic drugs primarily promote tumor necrosis and reduce blood flow perfusion rather than reducing tumor size [79]. Thus, vascular changes are more indicative of treatment effects than changes in tumor dimensions [80]. Additionally, these treatments often lead to phenotypic changes in liver lesions, such as necrosis, liquefaction, and hemorrhage, making size-based assessment alone insufficient and potentially underestimating the treatment response [81].

To overcome the limitations of the response evaluation criteria in solid tumors criteria, oncologists seek various parameters to evaluate tumor response to treatment. Chemotherapy, an essential treatment modality for advanced HCC, includes multi-kinase inhibitors, cytotoxic drugs, and molecularly targeted drugs [82]. In a study by Lv *et al*. [83] DECT imaging was used for the noninvasive quantitative monitoring of the therapeutic effect of vascular endothelial growth factor receptor inhibitors on rabbit VX2 liver tumors. There was a statistically significant correlation between the NIC differences of the tumor AP, portal phase, and the final change in the tumor size after 2 days of treatment. Conventional CT has been used to assess and monitor tumor response, but it provides few quantitative parameters and is insensitive or delayed in recognizing early response to therapy. DECT provides further data on the cancer microenvironment, which can be useful in the early assessment of response to therapy, especially in patients treated with anti-angiogenic drugs.

### 5.2 Assessment of Efficacy After Treatment with Arterial Chemoembolization

The early onset of HCC is often insidious, resulting in many patients being ineligible for radical surgery at the time of diagnosis, which leads to a poor prognosis. Transarterial Chemoembolization (TACE) is a common non-surgical treatment that involves blocking the blood flow to the tumor and injecting chemotherapy drugs [84]. However, many patients remain at risk of recurrence and metastasis after TACE treatment, often necessitating multiple treatments [85]. Therefore, timely and accurate evaluation of the effectiveness of TACE therapy and early detection of residual active lesions and recurrent metastatic lesions are of great significance for clinical treatment. Digital subtraction angiography is the most sensitive and specific method for assessing the efficacy of TACE [86]. Sincedigital subtraction angiography is an invasive test, it is not suitable for repeat monitoring. Consequently, postoperative evaluation by imaging, such as DECT, is crucial for monitoring efficacy and early detection of recurrence. Iodine mapping with DECT improves the visualization of ablation areas and margins and helps detect residual or recurrent tumors [87]. The level of iodine uptake can serve as a marker of tumor response after TACE treatment [88]. Choi *et al*. [89] found that lipiodol deposition in target lesions of HCC could be quantitatively analyzed by energy spectrum CT iodine to predict tumor response after TACE.

DECT parameters, especially λHU and NIC in AP, were effective in distinguishing different tissue components of HCC. The AUC values for identifying tumor active areas and adjacent normal liver parenchyma were 0.987 and 0.988, respectively, and the AUC values for identifying tumor active areas and tumor necrotic areas were 1.000 and 0.997, respectively [90]. Thaiss *et al*. [91] found that ID can be used as an effective perfusion marker to monitor local treatment response in HCC, possessing a diagnostic efficacy not inferior to that of perfusion CT while significantly reducing the radiation dose. Wang *et al*. [92] also found that NIC in AP had high sensitivity and specificity for predicting tumors with better iodine-oil deposition versus tumors without iodine-oil deposition and that patients with NIC values ≥ 0.18 in the AP of the lesion before TACE had a higher survival rate than patients with NIC values < 0.18 in the AP before TACE (P = 0.028). In addition, it is recommended to avoid the use of VNC images when evaluating the response after TACE. A study by Hur *et al*. [93] found that VNC images may produce false-positive or negative results due to imprecise deduction of iodine or iodized oil deposition, which can lead to discrepancies when compared to TNC images.

Gemstone spectral imaging allows for a more objective assessment of residual lesions and tumor recurrence or metastasis by comparing the spectral characteristics of suspected lesions with those of primary lesions. This enables gemstone spectral imaging to assess treatment efficacy more accurately and to detect active residual tumors and recurrent or metastatic lesions after TACE treatment [85].

### 5.3 Assessment of Efficacy After Local Ablation Therapy

Localized ablation of HCC, including Radiofrequency Ablation (RFA) and Microwave ablation (MW), is considered an appropriate treatment for Barcelona Clinic Liver Cancer stage 0 and A HCC [94]. Patients with HCC treated with local ablation have a high recurrence rate, making close postoperative monitoring essential. However, conventional imaging tools are often inadequate for assessing the efficacy of local ablation therapy.

Conventional CT is a commonly used imaging follow-up method after RFA in HCC patients, and conventional enhanced CT determines the efficacy of RFA based on the degree of previous lesion enhancement. With the advent of DECT, many DECT parameters have unique advantages for evaluating RFA efficacy. In the study by Li *et al*. [95], compared with the lesion before RFA treatment, the lesion Eff-Z, IC, and NIC showed a significant decrease after RFA treatment. The difference was statistically significant, which indicated that the arterial blood supply of the lesion was significantly reduced after RFA treatment, and the therapeutic effect was significant.

DECT can provide a more accurate quantitative analysis of liver tumors. Compared with traditional imaging methods, DECT can identify small changes in tumors more effectively, especially after RFA, and can show the perfusion and necrotic areas of tumors more clearly. This is of great significance in determining whether there is recurrence or metastasis of the tumor [96]. Lee *et al*. [97] used VNC images as an alternative to TNC images for efficacy assessment after RFA treatment for hepatocellular carcinoma. The overall image quality of VNC images was rated on a scale of 1-5, with smaller scores indicating higher image quality. The results showed that the VNC images were of good quality in 90% of the patients (mean score 1.88), and there was no significant difference between the mean CT values of the liver parenchyma and the ablation area between the TNC and VNC images (*P* > 0.05). The significance of lesions in the ablation zone on iodine maps was excellent or good in 97% of patients, and other benefits on ID maps were rated better or the same (mean score 1.5). CNR was significantly higher in aortic-hepatic parenchyma on ID maps (*P* = 0.002), and the difference in CT values between the center and periphery of the ablation zone was significantly lower (*P* < 0.001).

DECT-derived Volumetric Iodine Concentration (VIC) is a biomarker for imaging methods to assess HCC efficacy after MW, and VIC correlates moderately with pathological findings. It more accurately evaluates response to ablative therapy compared to conventional modified Response Evaluation Criteria in Solid Tumors (mRECIST) and Choi criteria [98]. The advantage of VIC is that it reflects the iodine uptake capacity of the tissue, avoids interferences in densitometric measurements caused by high-density coagulation or hemorrhage, and provides better reproducibility using software-automated volumetric measurements [99]. Additionally, VMI at 50 keV provides better image quality in the early assessment after MW [100, 101].

The potential value of IC parameters in predicting HCC progression within 12 months when evaluating HCC patients treated with RFA has been demonstrated [96]. Arterial iodine fraction and λHU, which can help differentiate tumor remnants from adjacent normal liver tissue, are valuable tools for predicting response after RFA treatment [90, 96].

## 6 OTHER APPLICATIONS OF DECT IN HCC

Compared with traditional CT, DECT has great breakthroughs in image quality details, substance composition determination and energy spectrum analysis. It is especially widely used in HCC, such as accurate diagnosis of HCC, evaluation of portal vein thrombosis, lymph node metastasis, and MVI and efficacy evaluation. In addition, DECT has shown potential in other applications of HCC diagnosis, such as in assessing the hemodynamic characteristics of HCC and providing more detailed information to help physicians develop personalized treatment plans. In HCC, there is a strong correlation between iodine density and arterial flow, and a later arterial phase (*i.e*., around 15-20 seconds after abdominal aorta enhancement) may be more suitable for detecting HCC in clinical practice [102]. Extracellular volume fraction and iodine density extracellular volume fraction of VMI were independent predictors of overall tumor survival and progression-free survival [103]. Analysis of energy spectrum curves helps to confirm the presence of enhancement in uncertain cases. The lesions with enhanced CT attenuation increased faster at lower energy levels (λHU steeper), while the curve with pseudoenhanced CT was flatter (λHU gentler) [66]. Combined imaging models of EDCT and perfusion CT predict macrotrabecular massive subtype, and cystic invasion in HCC. Studies have confirmed that among all tumor CT quantitative parameters, permeability surface area is the only important independent parameter for predicting recurrence risk [104]. Energy spectrum CT and perfusion CT can also be used to evaluate the blood supply source of PVT. IC and λHU at distal and proximal PVT and hepatic perfusion parameters were highly correlated with primary lesions, indicating that PVT was similar to primary liver lesions, both of which were mainly supplied by the hepatic artery [105].

In conclusion, the practical application of DECT in liver imaging shows extensive potential to provide important support for the diagnosis and management of liver diseases. As the technology continues to advance, DECT is expected to play a greater role in the field of liver imaging in the future.

## 7 RADIATION BURDEN AND COST-EFFECTIVENESS OF DECT

In the context of precision medicine, DECT provides a multiparameter, quantitative, and radiation-free examination method and has made significant progress in the diagnosis and efficacy assessment of liver cancer. In addition, DECT has shown promising performance in terms of radiation burden and cost-effectiveness.

Different parts of the body can be studied using contrast agents with different biodistribution properties, thus greatly reducing motion artifacts and radiation burden on the patient [7]. Nagayama *et al*. [106] introduced the DL-DECT method to minimize radiation exposure and iodine loading. It has been demonstrated that VMI at 50 KeV provides similar or higher image quality at similar radiation doses compared to conventional 80-100 kVp CT images [107]. A study by Lyu *et al*. [108] described an innovative approach aimed at reducing radiation exposure without compromising the quality of imaging through the use of low-dose DECT with DLIR algorithms.

The use of low-dose DECT-assisted DLIR resulted in a 34% reduction in radiation dose compared to full radiation dose Single-Energy Computed Tomography (SECT). DS-DECT allows the use of an automated exposure control system, which allows for more accurate radiation dose adjustments compared to fixed-tube current scanning used in KVS-DECT [109]. Similarly, the DS-DECT technique allows for radiation dose adjustment for different patient sizes, in contrast to the KVS-DECT technique [110]. In contrast, the DL-DECT technique utilizes a single X-ray tube and two layers of scintillation detectors. The inner layer captures low-energy photons, while the outer layer captures high-energy photons. This technique produces a radiation dose comparable to that of SECT [111].

In modern healthcare systems, cost-effectiveness analysis has become an important tool for assessing the economic value of medical interventions. It is especially important to conduct a comprehensive cost-effectiveness analysis in high-cost medical technologies such as DECT. DECT technology is gradually being widely used in clinical practice due to its advantages in imaging quality and diagnostic accuracy, but its high equipment and maintenance costs have raised concerns about its economics.

DECT can provide more accurate results than conventional CT in certain specific application scenarios, such as tumor detection and evaluation of cardiovascular disease. This accuracy not only improves the timeliness of diagnosis but also potentially reduces additional healthcare expenditures due to misdiagnosis. Studies have shown that DECT has better sensitivity and specificity than SECT in assessing myocardial perfusion and demonstrates better cost-effectiveness in terms of quality adjusted life years. For example, DECT has an incremental cost-benefit ratio of just $3,191 per quality-adjusted life years obtained, compared to $3,557 for single photon emission computed tomography, suggesting that DECT is economically superior [112]. Cost-benefit analyses of DECT for the diagnosis of occult hip fractures have shown that DECT provides higher quality-adjusted life years and an acceptable incremental cost-benefit ratio compared to conventional SECT and MRI [113].

## 8 LIMITATIONS AND PROSPECTS OF DECT

Since its introduction in 2006, DECT has evolved significantly, producing a wide range of derived sequences and providing more comprehensive imaging information for disease diagnosis. However, DECT is not perfect and still has some limitations. At the same time, with the continuous progress of science and technology and in-depth research, DECT also contains a broad development prospect.

DECT has unavoidable limitations in the diagnosis of HCC. Schmidt *et al*. [34] found that the accuracy of DECT-based iodine quantification is affected by the location of lesions near liver borders close to neighboring air. In addition, KVS-DECT has significantly more errors in the diagnosis of subdiaphragmatic lesions and an increased likelihood of artifacts compared to DS-DECT. Spectral separation is limited in a variety of DECT implementations, such as dual-layer detector and split filter systems, because a single multicolor beam results in the average energy of the two spectra used to calculate the decomposition of the material to be relatively close to that of the dual-source system, and the spectral overlap increases the image noise with measurement variability and raises the material's limit of detection, especially in patients with large body sizes. The minimum kVp spectrum of each DECT system is often limited to 70 or 80 kVp, below which threshold medium-sized adults are susceptible to photon starvation problems [114]. The 80/140 kVp combination is the most commonly used combination for DECT liver imaging and provides a good balance between soft tissue differentiation and image quality. However, this imaging modality may also have some limitations, such as large variations in pitch, rotation time, and tube current between different DECT techniques. The small second tube field of view of the DS-DECT scanner restricts its application to individuals with a high body mass index (often higher than 30 kg/m^2^) [109]. VNC image reconstruction is an important application of DECT. Although it can reduce the radiation dose and the number of imaging sessions, its clinical utility is limited by problems such as incomplete iodine subtraction and the subtraction of calcification and other substances.

In the diagnosis of HCC, DECT, as an emerging imaging technique, has shown some potential. However, there are still some limitations in its practical clinical application. Anzidei *et al*. [23] concluded that the VNC images of DECT can replace the TNC images to characterize HCC. In this study, 38 cases of HCC and 18 cases of benign liver lesions were identified with a diagnostic accuracy of 87.5 - 92.9%. However, the study also demonstrated that VNC images still have limitations, as 24.5% of small and dense lesions could not be accurately detected, and the use of VNC images as a substitute for TNC images would miss some lesions. The sensitivity of DECT for small liver lesions is about 69%, while its specificity is only 60% [47]. This means that in the early stages, DECT may not be effective in differentiating HCC from other benign lesions, leading to an increased risk of missed or misdiagnosis. In addition, image interpretation of DECT requires an experienced radiologist, as the results of image interpretation may vary depending on the experience and skill level of the reader [115]. Finally, the application of DECT is also affected by individual patient differences. For example, hepatic hemodynamic changes in patients with cirrhosis may affect the diagnostic performance of DECT [116]. Therefore, in clinical practice, doctors need to consider the specific situation of the patient as well as the results of other imaging tests to develop the best diagnostic strategy. Although DECT has certain advantages in the diagnosis of HCC, its clinical application still faces many challenges and limitations. Future studies are needed to further explore how to improve the sensitivity and specificity of DECT to better serve the diagnosis and treatment of HCC.

With the development of Artificial Intelligence (AI) technology, especially in IR, the application of AI offers new possibilities for DECT. In the future, AI, machine learning, and DL will play important roles in IR and optimizing DECT workflows [117, 118]. AI can significantly improve image quality and diagnostic accuracy, thus improving the diagnosis of HCC. The application of AI in DECT IR is mainly reflected in the DLIR technique. Compared with the traditional adaptive statistical iterative reconstruction, DLIR can effectively reduce the image noise and improve the signal-to-noise ratio and CNR, thus enhancing the image quality [118]. The use of this technique has allowed physicians to visualize lesion areas more clearly in liver imaging, improving detection rates and diagnostic confidence in liver lesions [119]. Second, AI can also segment and analyze liver images in an automated way. For example, using DL models such as convolutional neural networks, AI can automatically identify and segment the liver and its lesion areas. This automated processing not only improves work efficiency but also reduces the influence of human factors on the diagnostic results and ensures the consistency and accuracy of the diagnosis [120].

AI and its subsets, including machine learning and DL, also play an important role in optimizing the DECT workflow. DL algorithms reduce noise and artifacts to optimize the IR, and a certain KVS-DECT system uses the redundant information from low and high-energy datasets to generate a “Deep Learning View” and reconstructs and optimizes the DECT image [117]. With the rapid development of DECT technology and the development and application of AI-assisted one-button fast post-processing technology, DECT scanning will be further routinized, thus solving the limitations of current clinical practice such as the need to preset DECT scanning modes, spending a long time for post-processing, and the increase in the amount of image data, which is expected to maximize the advantages of DECT and apply it to the clinical workflow. Rich image and diagnostic information in the era of big data can further facilitate the rapid development of the AI field. AI shows great potential in IR and technology integration in DECT diagnosis of HCC. With the continuous advancement of technology and in-depth clinical application, AI is expected to play an even more important role in the diagnosis and therapeutic evaluation of HCC. In addition, in recent years, photon counting CT has continued to mature and gradually entered into clinical applications, and has shown its unique features and advantages, representing a revolutionary breakthrough in energy CT technology. With the deepening of clinical application, the future application of energy CT will surely have a broad prospect.

## CONCLUSION

In the past 10 years, the clinical application of DECT has become increasingly mature. With the advantages of high resolution and multiple quantitative parameters, DECT has brought a new direction for tumor imaging and diagnosis with satisfactory image quality and diagnostic performance and has played an important role in the diagnosis and treatment of HCC. With the continuous development and application of technology, DECT will achieve new breakthroughs in HCC grading, staging, and predicting metastasis in the foreseeable future.

## LIST OF ABBREVIATIONS

AI: Artificial Intelligence
AP: Arterial Phase
AUC: Area Under the Receiver Operating Characteristic Curve
CNR: Contrast-to-Noise Ratio
CT: Computed Tomography
DCNN: Deep Convolutional Neural Networks
DECT: Dual-Energy Computed Tomography
DL: Deep Learning
DL-DECT: Dual-Layer Detector Dual-Energy CT
DLIR: Deep-Learning Image Reconstruction
DS-DECT: Dual-Source Dual-Energy CT
Eff-Z: Effective Atomic Number
HCC: Hepatocellular Carcinoma
IC: Iodine Concentration
ID: Iodine Density
IR: Image Reconstruction
KVS-DECT: Rapid Kilovoltage-Peak Switching Dual-Energy CT
LNR: Lesion-to-Normal Parenchyma Iodine Ratio
MRI: Magnetic Resonance Imaging
MVI: Microvascular Invasion
MW: Microwave Ablation
NIC: Normalized Iodine Concentration
NID: Normalized Iodine Density
PVT: Portal Vein Thrombosis
RFA: Radiofrequency Ablation
ROI: Region of Interest
SECT: Single-energy computed tomography
TACE: Transarterial Chemoembolization
TNC: True Non-Contrast
VIC: Volumetric Iodine Concentration
VMI: Virtual Monochromatic Image
VNC: Virtual Non-Contrast-Enhanced
λHU: Slope of The Energy Spectrum
